# The demographics of patients affected by surgical disease in district hospitals in two sub-Saharan African countries: a retrospective descriptive analysis

**DOI:** 10.1186/s40064-015-1496-3

**Published:** 2015-12-01

**Authors:** Caris E. Grimes, Michael L. Billingsley, Anna J. Dare, Nigel Day, Peter M. George, Thaim B. Kamara, Nyengo C. Mkandawire, Andy Leather, Christopher B. D. Lavy

**Affiliations:** King’s Centre for Global Health, Weston Education Centre, King’s College London and King’s Health Partners, Cutcombe Road, London, SE5 9RJ UK; St George’s Hospital Medical School, University of London, London, UK; Oxford University Hospitals Trust, Oxford, UK; Bo Hospital, Bo, Sierra Leone; Port Loko Government Hospital, Port Loko, Sierra Leone; School of Community Health and Clinical Sciences, Njala University, Freetown, Sierra Leone; Connaught Hospital, Freetown, Sierra Leone; Department of Surgery, College of Medicine and Allied Health Sciences, Freetown, Sierra Leone; College of Medicine, University of Malawi, Mahatma Gandhi Road, Blantyre, Malawi; School of Medicine, Flinders University, Adelaide, Australia; University of Oxford, Oxford, UK

**Keywords:** Surgery, Low and middle income countries, Demographics, District hospitals

## Abstract

**Background:**

There is a growing awareness of the importance of surgical disease within global health. We hypothesised that surgical disease in low income countries predominantly affects young adults and may therefore have a significant economic impact.

**Methods:**

We retrospectively reviewed all surgical admission data from two rural government district hospitals in two different sub-Saharan African countries over a 6-month period. We analysed all surgical admissions with respect to patient demographics (age and gender), diagnosis, and procedure performed.

**Results:**

Surgical admissions accounted for 12.9 and 19.8 % of all hospital admissions in Malawi and Sierra Leone respectively. 18.5 and 6.2 % of all hospital patients required a surgical procedure in Malawi and Sierra Leone respectively, with the low number in Sierra Leone accounted for in that many of the obstetric admissions were referred to a nearby Medicins Sans Frontiers (MSF) hospital for treatment. 17.9 and 10.5 % of surgical admissions were under the age of 16 in Malawi and Sierra Leone respectively, with 16–35 year olds accounting for 57.3 % of surgical admissions in Sierra Leone and 53.5 % in Malawi. Men accounted for 53.7 and 46.0 % of surgical admissions in Sierra Leone and Malawi respectively. An unexpected finding was the high level of patients who absconded from hospital in Sierra Leone after diagnosis but before treatment. This involved 11.8 % of all surgical patients, including 38 % with a bowel obstruction, 39 % with peritonitis and 20 % with ectopic pregnancy.

**Conclusions:**

Most people affected by disease requiring surgery are young adults and this may have significant economic implications.

## Background

Surgery is increasingly being recognised as an important but neglected part of global health. An estimated 2 billion people worldwide have no access to surgical care (Weiser et al. 2008), with trauma alone accounting for nearly 10 % of all global mortality (Lozano et al. 2012). Furthermore, surgery has been shown to be cost-effective comparable to other routine public health interventions in low and middle-income countries (Grimes et al. 2014; Chao et al. 2014). However, much of the current data pertaining to the burden and consequences of surgical disease are based on best estimates, with many low income countries having no real data as to the burden and impact of surgery within their borders, particularly within rural areas. There is a need, therefore, to substantiate the understanding of global surgery with primary data from the ground.

There is a trend within global health research, policy and investment to understand not only mortality and morbidity rates of disease, but also the wider social and economic impacts of disease. This includes the demographic groups most affected (Pednekar et al. 2011; Addo et al. 2009; Damasceno et al. 2009; Vedanthan et al. 2014). The composition of emergency surgical admissions in high-income countries is predominantly disorders of the gallbladder, pancreas and liver, followed by colorectal disorders and upper gut. Almost 50 % of all emergency admissions in the United States are above the age of 60 (Gale et al. 2014). This contrasts significantly from demographic and disease patterns in low and middle-income countries where the population is young and conditions affecting childhood and early adulthood are still major contributors to the mortality figures. Therefore, it is perhaps even more essential that efforts are made to reduce the burden of disease on this important socio-economic group to limit the impact on national development and productivity.

The aim of this study was to describe the demographics of those affected by surgical disease by use of primary data from East and West Africa with the hypothesis, based on observation and anecdotal evidence, that surgical disease predominantly affects young adults.

## Methods

Two hospitals were selected for the project where the majority of clinical work and all surgical work is performed by locally trained staff. The hospitals covered similar population catchment areas and were within rural areas of East and West Africa respectively. Both hospitals provide some surgical care, and the logbooks were well maintained in order to conduct an analysis.

## Settings

### Thyolo District Hospital, Malawi

Malawi is a landlocked country in East Africa and has a population of approximately 15 million of which 85 % live in rural areas. Thyolo District Hospital in southern Malawi is a 350-bed government district hospital catering for a population of approximately 600,000. It has one fully qualified doctor, the District Medical Officer, who has the responsibility for the overall running of the hospital with the majority of the clinical work being performed by 20 clinical officers supported by nursing staff. It has two operating theatres, but only one that was in regular use during the period covered by this study. In Malawi, medical and surgical treatment is free at the point of delivery and patients therefore do not have to pay for equipment, patient stay, or any costs associated with their care including the operative procedure.

### Bo District Hospital, Sierra Leone

Sierra Leone in West Africa, has a population of approximately 6 million with 60 % of the population living in rural areas. Bo District Hospital in Sierra Leone is a 450 bed government district hospital catering for a population of approximately 600,000. It has two operating theatres, but at the time of data collection, only one was in use. Two MD trained medical officers provided surgical care, supported by a number of surgical nursing staff.

In Sierra Leone, although health care is free at the point of delivery for under 5’s, pregnant and lactating women, all other patients have to pay for costs related to bed stay, equipment, supplies, medications, investigations and the operation. The costs of the operation are divided into minor, intermediate and major procedures and are set centrally by the government each year.

### Data collection

Whole hospital inpatient data was collected retrospectively from two separate 3-month periods, representing rainy and dry seasons, through rigorous review and analysis of logbooks from all wards and theatres. The different sets of data were cross-referenced and, where possible, duplicates removed.

In Sierra Leone, all hospital data was collected for the months of July–September 2012 inclusive and February–April 2013 inclusive to capture both rainy and dry seasons. In Malawi, all hospital data was collected for the months of January–March 2013 and April–July 2013. As rainy and dry seasons differ in the two countries, the months studied were also different. The diagnoses were deemed “surgical” if the condition should be managed by a surgically trained provider. Any diagnosis, which was not recorded or not legible, was assumed not to be surgical to ensure conservative estimates. The list of “surgical” diagnoses is shown in Table 1. Analysis of the ward log books were used as the source for all surgical diagnoses, and analysis of both the ward logbooks and the theatre logbooks were used as the source for all surgical procedures performed.

**Table 1:** Surgical diagnoses included in study

Surgical diagnoses		
Abdominal pain Abscess Acute abdomen Anal fistula Animal bite Appendicitis Assault Basal skull mass Benign prostatic hyperplasia (BPH) Bowel obstruction Burn Cataract Cyst Diabetic foot Diabetic ulcer Dislocation Ectopic pregnancy Epididymo-orchitis Epistaxis Foreign body Fracture Haemorrhage Head injury Hepatic abscess Hernia Hydrocele Infected wound Injury	Keloid scar Lump Necrotising fascitiis Orchitis Osteomyelitis Otitis media Otitis sepsis Pancreatitis Paralysis Paraphymosis Penile fistula Peptic ulcer disease Peritonitis Priapism Pyomyositis Road traffic accident (RTA) Septic arthritis Septic finger Septic foot Septic sore Snake bite Soft tissue injury Solid organ tumour Stabbing Testicular torsion Trauma Unhappy triad of knee Urinary retention Wound	Complications of pregnancy including: Ectopic pregnancy Incomplete abortion Incomplete miscarriage Post-partum haemorrhage Prolonged labour Retained placenta Septic abortion

Because much of the obstetric surgical work is transferred from the government district hospital in Bo to the nearby Medicin Sans Frontiers (MSF) hospital at Gondama, we contacted MSF and obtained data from their records for the period studied for the district of Bo.

### Ethics approval

Ethics approval for data collection was obtained from the Malawi College of Medicine Research Ethics Committee and the Sierra Leone Ethics Committee.

## Results

### Surgical admissions as a proportion of total hospital admissions

In Thyolo District Hospital there were 6481 hospital admissions of which 835 (12.9 %) were surgical. In Bo District Hospital, there were 2152 hospital admissions of which 427 (19.8 %) were surgical. Not all surgical admissions required an operation. For example, patients with burns, head injury and assault were admitted into surgical wards, but many were managed conservatively. Conversely, not all patients who underwent a surgical procedure were admitted. For instance, the majority of patients undergoing evacuation of retained products of conception (ERPC) and some patients undergoing caesarean section at Thyolo were discharged home directly from the theatre complex. Analysis and comparison of the logbook data suggested that all other procedures were performed on patients also recorded on the inpatient data.

In Thyolo 898 patients underwent a surgical procedure in theatre. Analysis of the ERPC and caesarean section data showed that only 5 of the 332 patients who underwent an ERPC, and 271 of the 394 who underwent an emergency caesarean section, were admitted. Therefore the admission data does not capture many of the patients who underwent a procedure in theatre. If all patients who had undergone a procedure in theatre had been admitted, the total number of admissions would have been 6931 with 19.4 % of all hospital admissions being surgical and 18.5 % of all hospital patients requiring surgical input. With a population of approximately 600,000, the major operation rate for Thyolo was therefore 299 per 100,000 population per year.

In Bo, 133 patients underwent a surgical procedure. This corresponded to 6.2 % of all hospital admissions over the total 6 month period. As this is likely to be an underestimate as the majority of the maternal surgical work was performed at the nearby MSF hospital in Gondama, we contacted MSF to obtain their obstetric data for that time period. MSF data showed that over this 6 month period 296 caesarean sections were performed, 82 ERPC’s, 36 operations for ectopic pregnancy, 8 hysterectomies or oophorectomies and 8 other undefined obstetric or gynaecological procedures. 90.9 % of all obstetric and gynaecological procedures were performed on an urgent basis and 72 % were between the ages of 16–35 (Surgical Activity Reports 2013). Total operations (maternal and non-maternal) for the MSF hospital and for Bo District Hospital combined gave a procedure rate of 231 operations per 100,000 population per year (World Population Prospects 2012).

### Absconders in Bo

We recorded 33 (11.8 %) patients, having received a surgical diagnosis, subsequently absconded before receiving treatment. These included 3 of the 8 patients with bowel obstruction (38 %), 7 of the 18 patients with an acute abdomen (39 %) and 1 of the 5 ectopic pregnancies (20 %). This may have been because of the need to pay for hospital admission and any procedures undertaken, or it may be for sociocultural reasons.

### Age and gender distribution

**Fig. 1:**
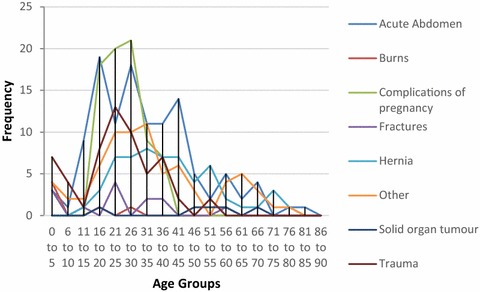
Diagnoses at different age groups, Bo District Hospital, Sierra Leone. Complications of pregnancy include breech presentation, pre-eclampsia, eclampsia, premature labour, prolonged labour, retained placenta, puerperal sepsis, post-partum haemorrhage, ectopic pregnancy, incomplete abortion, incompletely miscarriage. Acute Abdomen includes abdominal pain, peritonitis, peptic ulcer disease, appendicitis, bowel obstruction. Trauma includes head injury, road traffic accident, assault. Other includes hydrocele, snake bite, wounds, abscesses, urinary retention, anal/penile fistula, septic abrotion, pancreatitis, priapism, otitis media, diabetic foot/ulcer, septic finger, infected wounds, lumps, osteomyelitis, necrotising fasciiis, keloid scar, cyst, unhappy triad of knee, hepatic abscess

**Fig. 2:**
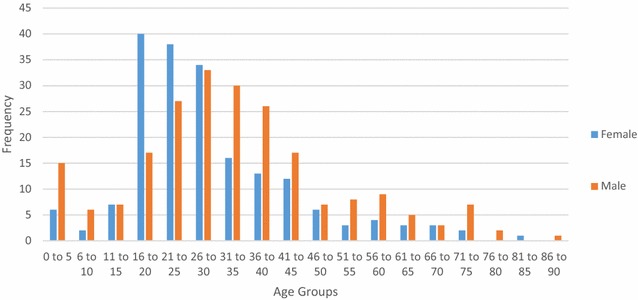
Gender distribution for diagnoses at Bo District Hospital

**Fig. 3:**
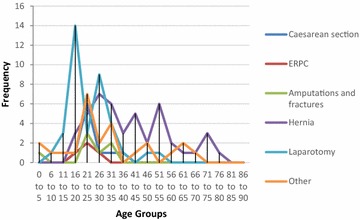
Procedures performed at different age groups, Bo District Hospital, Sierra Leone. Other: lump excision, hydrocele repair, wound management, abscess drainage. Laparotomy including appendicectomy

**Fig. 4:**
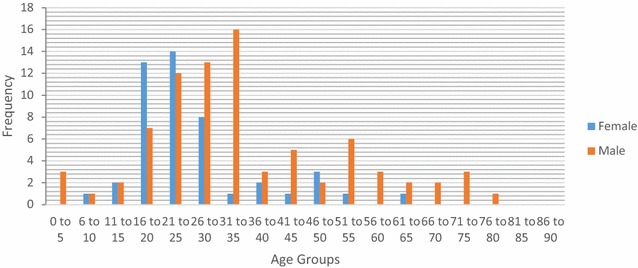
Gender distribution for surgical procedures at Bo District Hospital, Thyolo

**Fig. 5:**
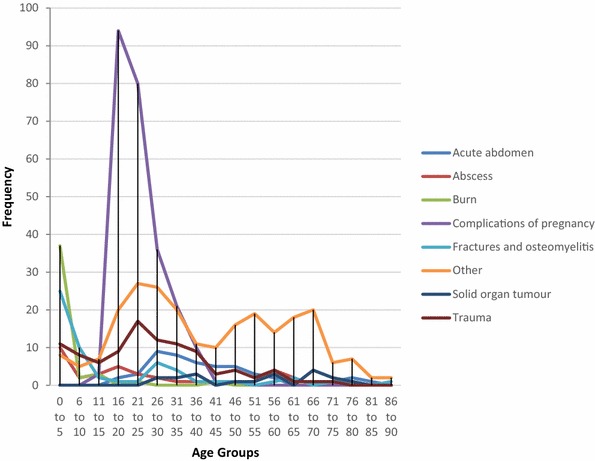
Diagnoses at different age groups, Thyolo District Hospital, Malawi. Complications of pregnancy including ectopic pregnancy and incomplete miscarriage. Acute abdomen: includes abdominal pain, pancreatitis, peptic ulcer, bowel obstruction. Trauma includes road traffic accident, stabbing, assault and injury. Other: Foreign body, septic sore, septic arthritis, wound problems, hydrocele, hernia, cataract, orchitis, epididymoorchitis, testicular torsion, paraphymosis, pyomyositis, animal bite, benign prostatic hyperplasia, lump, snake bite, soft tissue injury, basal skull mass, paralysis, otitis sepsis, epistaxis, haemorrhage

**Fig. 6:**
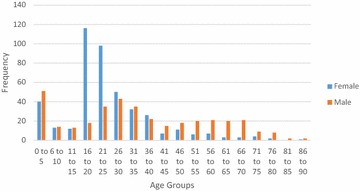
Gender distribution for diagnoses at Thyolo District Hospital

**Fig. 7:**
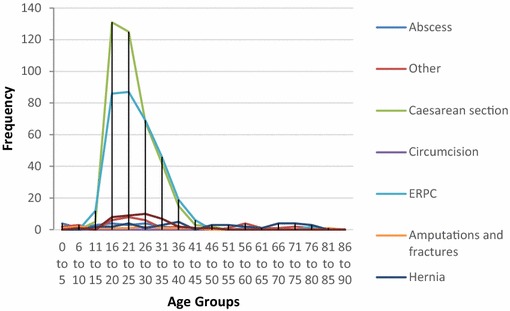
Procedures performed at different age groups, Thyolo District Hospital, Malawi. Other: biopsy, bilateral tubal ligation, lump excision, suturing of vaginal tear, examination of rectum under anaesthetic, prostatectomy, scrotal exploration foreign body removal, vaginal stenosis procedure

**Fig. 8:**
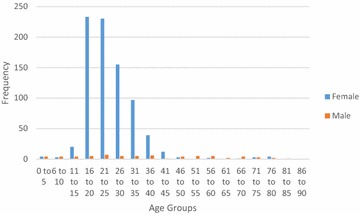
Gender distribution for procedures at Thyolo District Hospital, Malawi

Many surgical patients who were admitted did not require an operation. Conversely, some patients underwent a procedure but were not admitted. Therefore, we analysed surgical admissions and surgical procedures separately for Bo and Thyolo. The results are shown in Figs. 1, 2, 3, 4, 5, 6, 7 and 8. A number of patients were excluded from this analysis as there was no age recorded. The number excluded were 5 from the procedure data in Bo, 17 from the diagnostic data in Bo, 25 from the procedure data in Thyolo and 37 from the diagnostic data in Thyolo.

Analysis of the demographics revealed that under 16 year olds accounted for 10.5 % of surgical admissions in Bo, and 17.9 % of surgical admissions in Thyolo, with 16–35 year olds accounting for 57.3 % of all surgical admissions in Bo and 53.5 % of all surgical admissions in Thyolo. Men underwent 63 % of all surgical procedures in Bo but only 7.7 % of surgical procedures in Thyolo, because of the high rate of obstetric surgery in Thyolo which was not present in Bo.

**Fig. 9:**
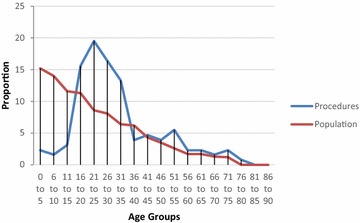
Procedures performed compared with population demographic in Bo District

**Fig. 10:**
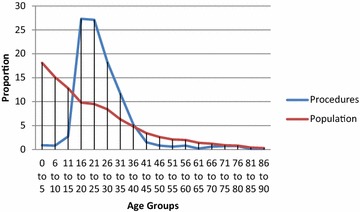
Procedures performed compared with population demographic in Malawi

As it was felt that the distribution of disease towards young adults may partly be due to a young population demographic, procedures were also plotted against the population demographics for Bo District Hospital and Malawi using census data (Population and Housing Census 2008; Census for Bo District 2015). The results, shown in Figs. 9 and 10, suggest that the young adult population treated at these hospitals cannot be accounted for by local population demographic alone.

## Discussion

### Comparison of Bo and Thyolo hospitals

Surgical conditions account for a significant burden of the district hospital work load in low income countries. This was quantified to be 19.4 and 19.8 % of all admissions for these two hospitals in two different countries in east and west Africa. The two hospitals in this study showed different types of procedures and volume based on the skills and facilities available. In Thyolo, few general surgical procedures were performed, but Thyolo did perform a large number of obstetric procedures which were not performed in Bo. This can largely be explained by local access to a larger centre specialising in areas of surgical practice. Conversely, in Bo, few maternal procedures were performed because of a local MSF hospital which offered these procedures free of charge. However Bo did have the facilities and skills to perform a large number of general surgical procedures, such as appendicectomies and non-gynaecological laparotomies which were not available in the same way in Thyolo.

Although the two hospitals volumes and procedures differed, the main age groups undergoing surgical treatment were similar. In both hospitals, just over half of all admissions were aged 16–35, with 10–17 % below the age of 16. This cannot be explained just by the age structure in these countries and is likely to be more reflective of the fact that many of these diseases such as appendicitis, obstructed labour, road traffic accidents, trauma and fractures are diseases of young adults. It is possible that young fit adults may be more likely to seek and attend for hospital care.

This study does not take into account outpatient surgical procedures. These would include incision and drainage of abscesses, suturing and/or debridement of wounds, reduction of dislocations and fractures, circumcision, removal of foreign bodies, child herniotomy etc. which would not routinely require admission.

### Surgical and non-surgical conditions

We chose to try and distinguish between “surgical” and “non-surgical” conditions for the purpose of this study in order to determine the demographics of surgical disease in district and rural government hospitals as a proportion of the total hospital workload. However, an analysis of inpatient data from the United States showed that surgical care cuts across the entire spectrum of disease categories, with no disease subcategory always requiring an operation, and no disease category that never required an operation (Rose et al. 2014). If this holds true for low income countries as well, it would imply that hospitals still need to have the facilities and skills of a surgical care provider to offer a comprehensive package of care for any given medical condition.

### Potential economic consequences

These two government district hospitals play a crucial role in averting death and disability from surgical disease in these countries. The economic impact of surgical disease is difficult to quantify and can be complicated to calculate but is likely to have a negative effect on average daily wage. According to the World Bank, the gross national income per capita for these countries is US $660 per annum for Sierra Leone and US $270 for Malawi (2013 figures) with life expectancy being 46 and 58 for men in these countries respectively. Untreated, a significant proportion of surgical disease results in death or disability. For example, a recent paper has suggested that if the rates of injury in low and middle income countries were reduced to those of high income countries, 2,117,500 lives could be saved each year with an economic saving of 758 and 786 billion dollars per year (Kotagal et al. 2014).

It has been shown that the health of a population impacts personal and national economic outcomes in several ways. Firstly, that healthy people are more likely to be employed and so there is a larger labour supply; secondly, that healthy people are likely to be more productive whilst at work; and thirdly, that healthy people are more likely to live longer, and in doing so, more likely to invest in their education (Thomson et al. 2009). Treating surgical disease, particularly as it predominantly affects the under-35 year olds, and may have a positive impact on personal and national wealth.

For example, as the economies of both Malawi and Sierra Leone are partly agricultural, it would be anticipated that an inability to use a limb would reduce the income to any given household and result in loss of earnings, reduction of household income, impoverishment and potentially reduction in GDP. For example, agriculture is thought to account for approximately 58 % of GDP in Sierra Leone and 36.1 % GDP in Malawi (African Development Bank 2010). Furthermore, disability in such settings usually removes a second family member from productive labour because of the need to care for the one who is disabled.

There is some evidence to demonstrate this point. Danquah et al. have shown that cataract surgery reduces disability and improves the economy of the household as well as health-related quality of life, an effect which is sustained 6 years after surgery (Danquah et al. 2014). It would be expected that treatments to prevent death from, for example, bowel perforations, intestinal obstruction, ectopic pregnancy; or treatments to reduce disability from, for example, fractures would have similar long term economic impacts.

### Unmet need and absconders

We estimated the met need to be 231 and 299 operations per 100,000 population per year in Sierra Leone and Malawi respectively. This is close to Weiser et al. estimate of 295 operations per 100,000 population per year (Weiser et al. 2008). In Sierra Leone, the total need has been estimated as 5200 operations per 100,000 per year (Hakon Bolkan, Capacare—unpublished) suggesting that the surgical need is only being met for 4 % of the Bo district population and this is likely to be similar for the Thyolo district population.

The estimation of unmet need in surgery (i.e. those who need treatment but do not obtain it) relies on population surveys of surgical conditions and verbal autopsy studies. A population based study of musculoskeletal impairment in Rwanda showed that the overall prevalence was 5.2 % of which 96 % required further treatment. 31.3 % were due to trauma and 3.8 % infection. 7.2 % were due to either the mal-union or non-union of fractures (Atijosan et al. 2008). A cross-sectional country wide survey in Sierra Leone suggested that 25 % of respondents reported a condition needing surgical attention and 25 % of deaths in the previous year may have been averted by timely surgical care (Groen et al. 2012).

The health system in Sierra Leone results in a number of patients being unable to afford the sometimes life-saving treatment that is required and an unknown number of patients abscond or fail to seek medical treatment as a result of this. Our study recorded only those that absconded after admission and therefore does not show the much greater unmet need of those who do not attend hospital in the first place.

Few other studies have addressed those who did not undertake surgical treatment because of costs. However, a study looking at elective surgery in rural Cameroon showed that of the 1213 patients presenting for pre-operative evaluation, 544 did not return for the operation after being told the costs of their treatment, leading to an estimated potential loss of 2163 DALYs. The most significant factor associated with failure to return for care was high costs of preoperative payment (Ilbawi et al. 2013). Men were more likely to return than women.

It would be thought, therefore, that if the skills, facilities and funding were available to treat all general surgical, orthopaedic and maternal health conditions at these two different hospitals, free of charge, then the number of surgically related admissions and procedure rates would rise and related death and disability reduced, with a likely resulting impact on personal and national wealth.

This hypothesis is of particular importance in low income countries such as Sierra Leone and Malawi. Public district hospitals are in some cases the only accessible form of modern healthcare to many of the population. It is for this reason that the surgical services must not be centralised to the biggest towns, out of reach for the majority of the population. These services must be available in the district hospitals as part of a broader development strategy, keeping the young and the primary contributors to the household, and indeed the national economy, fit and healthy.
